# Vitamin D Deficiency and Supplementation in Irritable Bowel Syndrome: Retrospective Evaluation of Subtype and Sex-Based Differences

**DOI:** 10.3390/medicina61071229

**Published:** 2025-07-07

**Authors:** Nur Düzen Oflas, Yonca Yılmaz Ürün

**Affiliations:** 1 Department of Internal Medicine, Van Yüzüncü Yıl University Medical Faculty, 65080 Van, Turkey; 2 Department of Gastroenterology and Hepatology, Van Yüzüncü Yıl University Medical Faculty, 65080 Van, Turkey; dryoncayilmazurun@gmail.com

**Keywords:** micronutrient imbalance, functional gastrointestinal disorders, sex differences, hematological parameters, biochemical parameters

## Abstract

*Background and Objectives:* Irritable bowel syndrome (IBS) is a prevalent functional gastrointestinal disorder with diverse subtypes. Recent evidence has suggested a link between vitamin D deficiency and IBS; however, the associations between vitamin D levels, IBS subtypes, and hematological–biochemical parameters remain unclear. The aim of this research was to investigate the associations between vitamin D status, IBS subtypes, and sex, along with their relationships with biochemical and hematological parameters. *Materials and Methods:* This retrospective study included 240 patients diagnosed with IBS according to the Rome IV criteria at Van Yüzüncü Yıl University Medical Faculty Hospital. The patients were classified as diarrhea-predominant (IBS-D), constipation-predominant (IBS-C), or mixed-type (IBS-M). The patients’ serum vitamin D levels and hematological (hemoglobin, white blood cell and platelet counts, and mean corpuscular volume) and biochemical (ferritin, iron, calcium, magnesium, and vitamin B12 levels) parameters were evaluated at baseline and after vitamin D supplementation. Sex-related differences were assessed. *Results:* Baseline vitamin D levels were low in all IBS subtypes, with no significant differences between the groups. Vitamin D supplementation resulted in a significant increase in serum vitamin D levels across all subtypes (*p* = 0.001). No significant correlations were identified between vitamin D levels and hematological or biochemical parameters. Sex differences in vitamin D levels were only significant in the IBS-M group, both at baseline and post-treatment (*p* < 0.05). *Conclusions:* Vitamin D deficiency is prevalent among all IBS subtypes and significantly improves with supplementation, independently of the subtype. Although no associations were found between vitamin D levels and laboratory parameters, the observed sex differences in patients with IBS-M highlight the need for further research into potential sex-related pathophysiological mechanisms. These findings support the integration of routine vitamin D assessment and supplementation into the clinical management of IBS, especially in patients with the IBS-M subtype and female sex, to potentially improve patient outcomes.

## 1. Introduction

Recent findings have expanded the known health advantages of vitamin D beyond musculoskeletal well-being, highlighting its potential connection to systemic diseases such as colorectal cancer and inflammatory bowel disease [1,2,3]. Vitamin D plays a vital role in regulating over 200 genes associated with the cell cycle, including proliferation, differentiation, and apoptosis [2,4]. It is well established that the vitamin D receptor (VDR) is expressed in a majority of tissues, including the gastrointestinal tract, nervous system, and immune cells [2,4]. Vitamin D is believed to affect irritable bowel syndrome (IBS) by playing a part in inflammatory processes, regulating the immune system, and modulating the gut microbiota. It might enhance the mucosal barrier and serve as an antimicrobial agent, which could help to alleviate the symptoms of IBS [5,6,7].

IBS is a functional disorder of the gastrointestinal system characterized by recurring abdominal discomfort and changes in digestive habits. The pathogenesis of IBS is multifaceted, involving the dysregulation of interactions between the brain and gut, along with various risk factors [8]. The prolonged nature of IBS demands significant medical resources, greatly impacting the quality of life of both patients and their families and imposing a substantial societal burden [9,10]. Considering the significant burden of IBS, it is essential to deepen our understanding of its pathological mechanisms to develop effective treatment strategies [11]. The signaling pathways associated with vitamin D and its receptor play a crucial role in the immunological, genetic, environmental, and microbial dimensions of human health and disease [12]. Numerous investigations have elucidated the essential role of vitamin D and its receptor in the preservation of optimal gastrointestinal health [13,14]. Approximately 15.7% of the global population is affected by vitamin D deficiency and 47.9% by vitamin D insufficiency, with a slightly higher prevalence among individuals with IBS [15,16].

Given the presence of VDRs in the gastrointestinal and nervous systems, and its role in immune modulation, a growing body of literature has hypothesized a potential pathophysiological link between vitamin D deficiency and IBS symptoms. Although vitamin D deficiency is prevalent among patients with IBS, the association between vitamin D levels and the severity of gastrointestinal symptoms in these patients remains unclear [17,18]. Previous studies have explored the association between vitamin D deficiency and IBS, and the findings remain inconsistent, particularly regarding the distribution of vitamin D deficiency across IBS subtypes and the role of sex-based differences. Additionally, data on the relationship between vitamin D levels and routine hematological and biochemical parameters in IBS populations are limited. Given these gaps, a retrospective observational study could provide valuable insights by analyzing real-world clinical data. This study aimed to explore the associations between vitamin D status, IBS subtypes, and sex, as well as their relationships with biochemical (ferritin, iron, calcium, magnesium, and vitamin B12 levels) and hematological (hemoglobin, white blood cell and platelet counts, and mean corpuscular volume (MCV)) parameters. By addressing these questions, this study contributes to the existing literature by clarifying the distribution patterns of vitamin D deficiency in IBS and identifying potential subgroup-specific considerations for its clinical evaluation. We hypothesized that vitamin D deficiency is prevalent across all IBS subtypes and that vitamin D supplementation would significantly increase serum vitamin D levels, regardless of the subtype. Furthermore, we hypothesized that sex-based differences might influence baseline levels and responses to supplementation, particularly in the IBS-M group.

## 2. Materials and Methods

**Figure 1:**
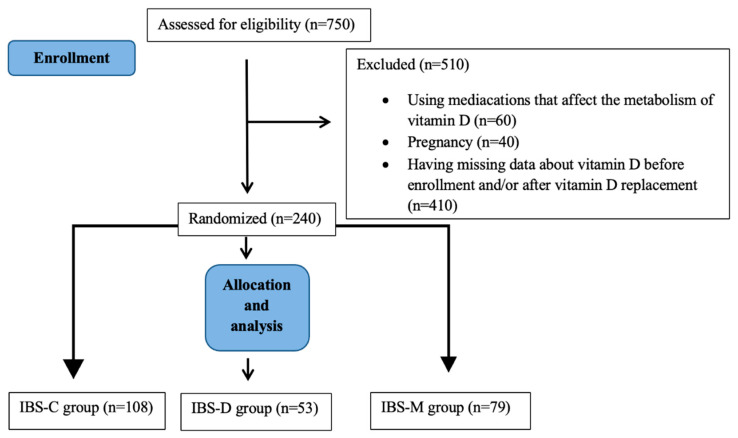
Flow chart of patient inclusion. A flow diagram illustrating the selection process of patients diagnosed with irritable bowel syndrome (IBS) based on the Rome IV criteria. Of the initial 750 patients screened from the hospital database, 240 were included in the final analysis after the application of inclusion and exclusion criteria, such as age (18–85 years), medication history, and availability of complete biochemical records.

For this retrospective cohort study, patients with IBS who were followed up and treated at the Van Yüzüncü Yıl University Faculty of Medicine Hospital between January 2020 and December 2024 were retrospectively screened for this study. The patients’ data were obtained by an internal medicine specialist scanning their electronic medical records from the hospital database. The inclusion criteria were as follows: aged between 18 and 85 years; not using medications that affect the metabolism of vitamin D, such as seizure drugs or anti-tuberculosis drugs; not pregnant or lactating; and free of active infections. The exclusion criteria were as follows: age below 18 years, use of medications that affect vitamin D metabolism, pregnancy and/or breastfeeding, and active infections. A total of 750 IBS patients were screened retrospectively from their medical records. After the exclusion criteria were applied, 240 patients were included in this study. The participant inclusion process is illustrated in Figure 1. The Rome IV diagnostic criteria were used to diagnose patients with IBS; these included recurrent abdominal pain on an average of at least 1 day/week in the last 3 months, associated with two or more of the following criteria: (1) related to defecation, (2) associated with a change in the frequency of stool, and (3) associated with a change in the form (appearance) of stool. These criteria should be fulfilled in the last 3 months, with symptom onset at least 6 months before diagnosis. The IBS patients were categorized into three subtypes: (1) constipation-predominant (IBS-C), (2) diarrhea-predominant (IBS-D), and (3) mixed-type (IBS-M) [19]. Ethical approval was obtained from the Ethics Committee of the Van Yüzüncü Yıl University Medical Faculty (decision date: 04.02.2025; No. 2025/01-35). This study was conducted in accordance with the guidelines of the Declaration of Helsinki (revised in 2013). The requirement for informed consent was waived owing to the retrospective design of this study. This retrospective cohort study was conducted and reported in accordance with the Strengthening the Reporting of Observational Studies in Epidemiology (STROBE) guidelines. Venous blood samples were collected from each patient. For the hemogram examination, samples taken in EDTA tubes were stored under appropriate conditions and tested using a Mindray BC 6800 hemogram analyzer. For biochemical tests, serum samples were taken in a yellow tube containing gel. The samples were left for 15 min to clot, then centrifuged at 4000 rpm for 10 min. The serum was then separated and analyzed using a Beckman AU 5800 autoanalyzer. From the hemograms, measurements of hemoglobin, MCVs, platelet counts, and leukocyte counts were documented and analyzed. Concurrently, biochemical assessments were performed, including the documentation and analysis of vitamin B12, vitamin D, calcium, magnesium, iron, and ferritin levels. Vitamin D supplementation was provided to the patients at a dose of 50,000 IU weekly for a period of eight weeks.

### Statistical Analysis

Statistical analyses were performed using Analyze-it for Microsoft Excel (Analyze-it Software, Ltd., Leeds, UK). Quantitative variables are presented as means and standard deviations or medians and percentiles (25–75%), depending on whether they showed a normal distribution. Data were tested for normality using the Shapiro–Wilk test. The Chi-square test was used to examine the relationships between categorical data. Group comparisons were performed using a one-way ANOVA for continuous data with a normal distribution. Group comparisons were performed using the Kruskal–Wallis test for continuous data without a normal distribution. A paired t-test was used to compare the differences between repeated measurements. A post hoc power analysis was conducted based on the change in serum vitamin D levels before and after supplementation. Using the paired t-test model, with an observed effect size (Cohen’s d) of approximately 0.9 (based on the mean increase from 12.0 ng/mL to 32.0 ng/mL with SD ≈ 10), a minimum of 34 subjects would be required to achieve a power of 0.80 at a significance level of 0.05. The threshold for statistical significance was set at 0.05.

## 3. Results

A total of 240 patients were included in this study. The median age was 44 (35–57) years in the IBS-D group, 46 (37–60) years in the IBS-C group, and 44 (30–61) years in the IBS-M group. Of all the patients, 47.5% were female. According to the Rome IV criteria, among these 240 patients with IBS, 22% had IBS-D, 45% had IBS-C, and 33% had IBS-M. The other demographic and biochemical characteristics are summarized in Table 1. As summarized in Table 1, no significant differences were observed among IBS subtypes with respect to demographic, hematological, or biochemical parameters, including baseline and post-treatment vitamin D levels. All subgroups exhibited similarly low vitamin D levels at baseline, and reacted similarly to supplementation as well.

**Figure 2:**
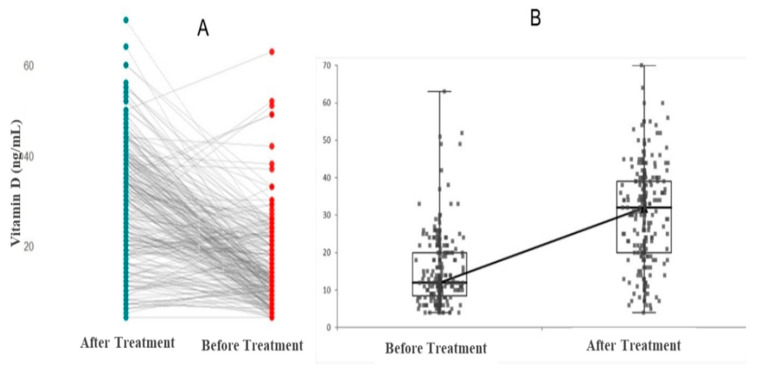
Changes in vitamin D levels before and after supplementation. (**A**) Individual patient trajectories demonstrating serum vitamin D levels before and after two months of supplementation. (**B**) Box-and-whisker plots displaying the distribution of vitamin D levels pre- and post-treatment. A significant increase in serum vitamin D concentration was observed across all IBS subtypes following supplementation (*p* = 0.001).

The impact of treatment with a vitamin D supplement on serum vitamin D levels was assessed using both individual and summary visualizations. As shown in Figure 2A, most patient trajectories showed an upward trend, suggesting an overall increase in vitamin D levels after the intervention. This trend is reinforced by the box-and-whisker plot in Figure 2B. Specifically, the median serum vitamin D level increased from 12.0 ng/mL (25th–75th percentiles: 8.42 to 20.0) before treatment to 32.0 ng/mL (25th–75th percentiles: 20.0 to 39.0) after treatment (*p* = 0.001). When stratified by IBS subtype, all patient groups exhibited a significant increase in serum vitamin D levels after treatment (Table 2).

**Figure 3:**
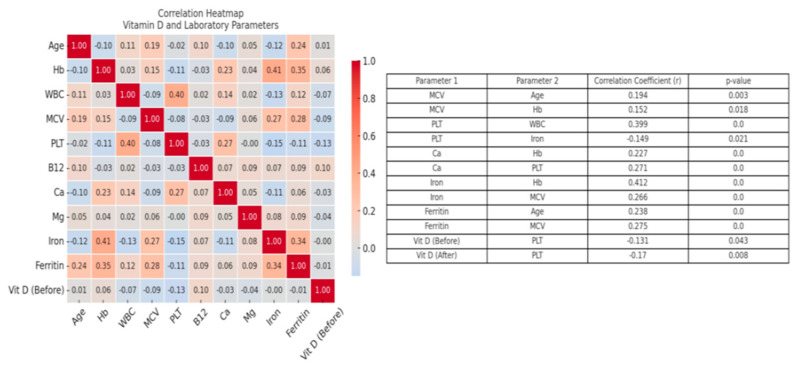
Correlation analysis of baseline vitamin D levels with biochemical and hematological parameters. Scatter plots showing the absence of significant linear correlations between baseline serum vitamin D levels and selected biochemical (calcium, magnesium, ferritin, iron, vitamin B12) and hematological (hemoglobin, WBC, platelet count, MCV) markers. Pearson correlation coefficients were calculated, with all *p*-values > 0.05.

A correlation analysis was performed to explore the potential associations between baseline serum vitamin D levels and various hematological and biochemical parameters. As shown in Figure 3, no statistically significant correlation was observed between vitamin D levels and age, hemoglobin, white blood cell count, platelet count, MCV, or common micronutrients, such as calcium, magnesium, iron, or ferritin (all *p* > 0.05).

When stratified by both sex and IBS subtype, in the IBS-M group, vitamin D levels differed significantly between the sexes at both time points (Table 3).

## 4. Discussion

In this study, we investigated the relationship between vitamin D status, IBS subtypes, and sex-based differences, as well as the associations with routine laboratory parameters. Our results showed that vitamin D deficiency was prevalent across all IBS subtypes, improving significantly following vitamin D supplementation. However, no significant correlations were found between vitamin D levels and routine hematological or biochemical markers. Notably, the serum vitamin D levels of women in the IBS-M group were found to be significantly lower than those of men, both before and after treatment. This suggests an interaction between sex and IBS subtype.

Although there is much evidence suggesting a link between vitamin D deficiency and IBS, the exact nature of this relationship is not fully understood. It has been suggested that vitamin D may play a role in the pathophysiology of IBS by regulating the immune system, controlling inflammation, and modulating the gut microbiota [13]. Although numerous studies have reported positive correlations, some have not identified a notable difference in vitamin D levels between individuals with IBS and healthy individuals. For example, Ali et al. studied the correlation between vitamin D deficiency and IBS in 90 patients (45 healthy individuals vs. 45 IBS patients). They found that the prevalence of vitamin D deficiency was higher in the IBS group (76.6% vs. 46.7%) [5]. Similarly, in a study conducted by Husseiny et al., 56.06% of patients with IBS had vitamin D deficiency, while 39.65% had insufficient vitamin D levels [20]. In contrast, a study conducted by Matthews et al. reported no significant association between plasma vitamin D concentrations and IBS symptoms in women. In their study’s IBS group, 40.3% were vitamin D-deficient and 33.9% were vitamin D-insufficient. The corresponding rates were similar in the healthy control group, with 41.4% deficient and 34.3% insufficient [21]. Similarly, Abuelazm et al. revealed that vitamin D supplementation failed to improve IBS symptoms [22]. In our study, vitamin D levels were found to be low at baseline and increased significantly in both men and women with all IBS subtypes following replacement therapy. However, not all studies have demonstrated consistent benefits, potentially due to methodological differences, such as variations in patient populations, supplementation protocols, geographic location or outcome measures. For instance, studies that incorporate symptom-based outcomes may reach different conclusions to those of our study, which focused on biochemical parameters.

Besides this, we observed that women generally had lower vitamin D levels, both before and after treatment, than men, although this difference was statistically significant only in the IBS-M group. The fact that this difference was only observed in IBS-M suggests that different subtypes may be associated with different pathophysiological mechanisms, that there may be sex-specific differences in the metabolism or absorption of vitamin D, and that these differences are more pronounced in patients with IBS-M. Typically, women have lower levels of vitamin D than men, which is attributed to a mix of biological, lifestyle, and environmental influences. Variations in body composition, hormonal differences, and cultural customs that influence sun exposure and eating patterns contribute to these distinctions [23,24,25]. The molecular mechanisms underlying the differences in serum 25(OH)D concentrations between men and women remain unclear. Recent observations have shown that the use of estrogen-containing contraceptives leads to an increase in 25(OH)D levels [26]. Women have higher levels of vitamin D-binding protein (DBP), which is associated with estrogen-dependent DBP production, which may reduce the availability of free, biologically active vitamin D [27]. Certainly, sex hormones play a crucial role in influencing the gene expression levels related to vitamin D. However, they may not be the sole factor. Therefore, alternative mechanisms may account for these differences.

In a study of healthy individuals with vitamin D deficiency, Firmicutes (55.86%) and Bacteroidetes (40.70%) were found to be dominant in their microbiota compositions, accounting for 95%, with Firmicutes being more prevalent. A decrease in Firmicute density and an increase in Bacteroidota density were observed following vitamin D replacement therapy [28]. The presence of Firmicutes in the gut microbiota can influence the expression of VDR target genes in the colon, which are crucial for the regulation of vitamin D metabolism. This modulation is linked to the production of bile acids by Firmicutes, which can activate the VDR and affect downstream vitamin D signaling pathways [29]. A study by Gryaznova et al. revealed statistically significant differences, characterized by an increase in the abundance of the phyla Firmicutes, Actinobacteria, and Verrucomicrobia, and a decrease in the abundance of the phylum Bacteroidetes, in IBS-C and IBS-M groups compared to healthy controls [30]. A comparative analysis revealed that the relative abundances of Firmicutes and Actinobacteria exhibited a notable increase, whereas the abundance of Bacteroidetes demonstrated a significant decline in females in contrast to males [31]. This may explain why vitamin D levels were found to be significantly lower in women than in men, both before and after replacement therapy, for the IBS-M subtype in our study. Although there is limited direct evidence specifically addressing sex differences in vitamin D status within IBS-M patients, the proposed mechanisms offer biologically plausible explanations based on existing literature and require further investigation in prospective studies. Future research should explore whether this finding is due to hormonal, genetic, or microbiome-related mechanisms, as understanding this could highlight subgroups of IBS patients (e.g., women with IBS-M) who might benefit from tailored management strategies.

Besides the vitamin D-related findings, we also examined the distribution of IBS subtypes in our sample relative to the literature. In our cohort, IBS-C was the most common subtype (45% of patients), whereas some reports from other populations (e.g., a U.S. study) have identified IBS-M as slightly more prevalent [32]. This discrepancy may be influenced by the demographic characteristics of our sample—particularly the female predominance—since women are often reported to have IBS-C more frequently, while men have higher rates of IBS-D or IBS-M [33]. In our data, the subtype distribution difference by sex did not reach statistical significance, likely due to the sample size and retrospective design. Nonetheless, the trend aligns with known sex-related patterns in IBS, which have been attributed to differences in gastrointestinal motility, hormonal factors, and gut–brain interactions between men and women [34,35]. This context underscores that sex is an important factor in IBS research and might intersect with other variables like vitamin D status, as seen in our IBS-M subgroup results.

Our study revealed no statistically significant differences in sex distribution; hematological parameters such as hemoglobin levels, white blood cell counts, MCVs, and platelet counts; or biochemical markers such as levels of essential micronutrients, including calcium, magnesium, iron, and ferritin, among the IBS subtypes. Ergenç et al. found significant relationships between vitamin D levels and several hematological parameters, including platelet counts, monocyte counts, MCVs, neutrophil counts, and calcium and vitamin B12 levels. Platelet and monocyte counts were significantly higher in the group with low vitamin D levels (<20 ng/mL). However, the neutrophil counts, MCVs, and calcium and B12 levels were significantly lower in this group [36]. Mobarki et al. investigated the prevalence of vitamin D deficiency among young adults and its correlation with various hematological and biochemical parameters. Their study found no significant differences in hematological parameters (white blood cells, platelets, and red blood cells) among participants based on their vitamin D status [37]. Although this study did not identify significant correlations between vitamin D levels and common laboratory parameters such as hemoglobin, ferritin, magnesium, or inflammatory markers, the findings may reflect the complexity of vitamin D’s role in systemic and gastrointestinal physiology. Vitamin D has been shown to influence gut barrier integrity, modulate mucosal immunity, and affect microbial composition—none of which are captured by conventional serum markers [38]. Prior studies have reported inconsistent relationships between vitamin D levels and various biochemical or inflammatory markers, suggesting that its immunomodulatory effects may be more functional than purely quantitative [39]. Moreover, growing evidence supports a critical role of vitamin D in the gut–brain axis, particularly in functional gastrointestinal disorders such as IBS. It is thought to influence neuroimmune signaling, reduce visceral hypersensitivity, and improve central pain modulation [38]. These mechanisms may contribute to symptom relief in IBS independently of changes in standard laboratory values. Thus, while our study did not demonstrate biochemical correlations, this does not preclude potential clinical benefits of vitamin D supplementation in IBS, particularly in symptom improvement and quality of life—areas that future interventional studies should target.

In summary, the findings of this study suggest that routine screening for vitamin D deficiency in IBS patients, particularly those with the IBS-M subtype and of female gender, could be clinically valuable. Given the high prevalence of deficiency and favorable response to supplementation, integrating vitamin D assessment into the IBS diagnostic process could be a cost-effective, non-invasive way of addressing potential contributing factors. This is particularly relevant in settings where access to advanced investigations is limited. Furthermore, patients with refractory or fluctuating symptoms despite standard management may benefit from evaluating and correcting their vitamin D levels as part of a holistic treatment plan.

Our study had some limitations. The retrospective design, selected patient population, and the fact that the data were from a single center limit the generalizability of the study findings. The failure to evaluate biomarkers potentially associated with vitamin D, such as inflammatory markers and microbiota composition, is another limitation of our study. Furthermore, due to this study’s retrospective design, selection and information bias cannot be entirely ruled out. Despite the use of standardized inclusion criteria and the inclusion of all eligible cases within the defined period, variability in medical record documentation and the single-center nature of the cohort may have introduced inherent biases. Finally, symptom scores and quality-of-life measures were not assessed after vitamin D replacement. Therefore, the clinical implications of these biochemical improvements should be evaluated in future prospective studies.

## 5. Conclusions

In conclusion, this study demonstrated that vitamin D deficiency is highly prevalent among patients with irritable bowel syndrome and that vitamin D supplementation leads to significant improvements in serum levels across all subtypes. While no direct correlations were found between vitamin D and routine biochemical or hematological markers, the identification of a statistically significant sex-based difference in the IBS-M group highlights a potential avenue for personalized treatment strategies. These findings support the routine assessment and correction of vitamin D deficiency as part of the clinical management of IBS. Furthermore, this work contributes to the emerging literature on micronutrient modulation in functional gastrointestinal disorders and advocates for future prospective studies to clarify causal mechanisms and therapeutic outcomes. Importantly, future studies should investigate whether vitamin D supplementation not only improves biochemical profiles, but also alleviates gastrointestinal symptoms and enhances quality of life in patients with IBS. The identification of sex differences within the IBS-M group may introduce a new topic for discussion in the literature, warranting consideration for future research.

## Figures and Tables

**Table 1:** Comparison of biochemical and hematological variables and demographic characteristics among irritable bowel syndrome subgroups.

	IBS-D * Group (N = 53)	IBS-C * Group (N = 108)	IBS-M * Group (N = 79)	*p* Value
Demographic Characteristics				
Sex				
Female	40	74	45	0.488
Male	13	34	34	
Age (years)	44 (35–57)	46 (37–60)	44 (30–61)	0.598
Hematological Parameters				
Hemoglobin (g/dL)	14.2 (13.1–15.9)	14.4 (13.4–15.8)	14.4 (13.2–15.7)	0.728
White blood cell (×10^3^)	8.0 (6.59–8.89)	7.3 (6.2–8.7)	7.3 (6.0–9.3)	0.114
Mean corpuscular volume (fL)	84.8 (81.6–88.0)	84.9 (83.1–89.8)	85.9 (83.1–88.0)	0.357
Biochemical Parameters				
Platelet (×10^3^)	294 (235–336)	274 (235–316)	280 (237–327)	0.579
Calcium (mg/dL)	9.53 (8.91–9.81)	9.56 (9.21–9.80)	9.60 (9.20–9.90)	0.498
Magnesium (mg/dL)	1.96 (1.84–2.11)	1.95 (1.85–2.11)	1.93 (1.80–2.08)	0.609
Iron (μg/dL)	74.1 (51.6–94.1)	78.3 (58.3–103)	76.0 (52.0–96.6)	0.659
Ferritin (ng/mL)	48.5 (18.8–85.3)	50.6 (18.8–98.2)	55.3 (23.0–121.8)	0.082
Vitamin Levels				
Vitamin B12 (pg/mL)	277 (234–378)	315 (241–389)	280 (237–327)	0.163
Vitamin D level at admission (ng/mL)	12.0 (10.0–20.0)	13.0 (9.01–22.0)	11.0 (8.00–40)	0.193
Vitamin D level after 2 months (ng/mL)	33.0 (26.0–36.0)	32.0 (20.0–40.0)	28.0 (18.0–40.0)	0.652

* IBS-D: diarrhea-predominant IBS; IBS-C: constipation-predominant IBS; IBS-M: mixed-type IBS.

**Table 2:** Comparison of serum vitamin D levels before and after treatment in IBS subgroups.

	IBS-D * Group (N = 53)		
	Before Treatment	After Treatment	*p* Value
Vitamin D (ng/mL)	12 (10–20)	33 (25–36)	0.001
	IBS-C * Group (N = 108)		
Vitamin D (ng/mL)	13 (9–22)	32 (20–40)	0.001
	IBS-M * Group (N = 79)		
Vitamin D (ng/mL)	11 (8–18)	28 (18–40)	0.001

* IBS-D: diarrhea-predominant IBS; IBS-C: constipation-predominant IBS; IBS-M: mixed-type IBS.

**Table 3:** Comparison of serum vitamin D levels between female and male patients with different IBS subtypes before and after treatment.

	Female	Male	*p* Value
	IBS-D * Group (N = 53)		
Before Vitamin D treatment (ng/mL)	15 (10.2–20)	11 (7.51–16.5)	0.291
After Vitamin D treatment (ng/mL)	33 (21.2–33.7)	34 (26.5–39.5)	0.223
	IBS-C * Group (N = 108)		
Before Vitamin D treatment (ng/mL)	12 (8.75–20)	16 (11–23.5)	0.150
After Vitamin D treatment (ng/mL)	32 (19–40)	32.5 (20–40.5)	0.372
	IBS-M * Group (N = 79)		
Before Vitamin D treatment (ng/mL)	10 (7–13.5)	13.5 (8–22.5)	0.025
After Vitamin D treatment (ng/mL)	26 (16–39)	34 (18.7–40.5)	0.03

* IBS-D: diarrhea-predominant IBS; IBS-C: constipation-predominant IBS; IBS-M: mixed-type IBS.

## Data Availability

The original contributions presented in this study are included in the article; further inquiries can be directed to the corresponding authors.
